# Religion and the Housing Question

**Published:** 1919-03-01

**Authors:** 


### THE GRAVE SCANDAL OF BETHNAL GREEN.

### RELIGION AND THE HOUSING QUESTION.

### By A SOCIAL WORKER.

THE faith of Christians, if not the Christian Faith itself, is on its trial before the world's judgment. Lately a Mahometan saw in the fact that Christians had established a hospital in which they were nursing the enemies of their faith, his co-religionists, a testimony to the grandeur of their creed. But the missionary to whose arguments an Indian pundit simply said, "I have been in Whitechapel," had a sadder tale to tell. Against that argument from experience no preaching could hope to make way. The demand is for a revival of religion, but of *applied* religion, the religion of St. James. Nothing is more painfully striking to those of us whose eyes have been opened, if only a little, to the tragedy of civilisation than to read now any of the memoirs of pious people of the last two centuries, with the exception of the few great workers and reformers. While they read their Bibles and prayed and fought against evil thoughts, and aspired and toiled to acquire virtues, the slum was growing up. The great industrial revolution speeded the growth of towns, education in any real sense was discouraged as unfitting "the lower orders" for their place in the world, and the distinction between the healthy simplicity of cottier life and the inhuman misery of the slum was forgotten, or rather never learnt.

How should it be? "When the eye does not see, the heart does not grieve." Kingsley wrote *Alton Locke*, and some worthy people at least fought shy of the Radical Parson. There came a later day when settlements were founded, and to go "slumming" became fashionable. It was a passing fashion, but efforts of all kinds began and increased; and, as usual in England, the work of volunteers pointed to Governments the way of duty. National health was seen to be a necessity; the war came, and threw a new and lurid light on the conditions of town and village. We owe to it the cry for "Homes fit for Heroes to live in."

### Will London Awake?

Moreover, we have perceived with dismay that you cannot with impunity leave a festering sore in a body, or a rotting bruise in a fruit or a slum in town and keep fruit or body or city in a healthy state. For four years we have been told that so soon as peace came this evil would at once be swept away. *Will it?* For the first time probably in the history of the Church of England, an indignation meeting was solemnly held in a church to protest against any delay in the removal of this terrible evil, and was addressed by laymen. There was no applause, no discussion, no collection, no pledge asked at St. Martin's, Charing Cross Road, on Saturday, February 22, but the plain facts of the position in Bethnal Green were put before the large congregation admitted by free tickets, and these facts were illustrated by lantern-slides. The speakers were the Mayor of Bethnal Green, Councillor Edmonds, its representative on the L.C.C., and Mr. Douglas Eyre, of Oxford House, with the Bishop of Stepney to replace Mr. Sheppard, absent through illness.

But the plain unvarnished tale hardly needed the impassioned appeal made by Mr. Edmonds to those present to consider what they individually could do for these, their fellow-citizens and fellow-Christians, or, for that matter, for their own families in friends whose lives and whose health are indirectly threatened by the infamous conditions. Is not some of the most high-class tailoring in London done in Bethnal Green? Do not the tuberculosis germs grown in that admirably adapted hot-bed flow with every breeze through London? Consider the facts.

**In a given area there are 1,788 houses admittedly unfit for human habitation in 92 so-called streets. Some of these streets are passages 9 feet wide. Some of these houses are of bricks so rotten that they crumble to powder when rubbed with a stick. Nay, more, in some cases a walking-stick can be pushed right through the mouldering wall.**

The minimum standard of healthy housing is put at fifty-five persons to an acre. There are three and a quarter acres here which house 950 persons on an acre. There are back-to-back houses, illegal for nine years past to build. But these and other condemned houses have been, after official closing, tinkered by landlords, and by permission re-let. Some have absolutely no sanitation. Back-to-back houses, of course, have no ventilation throughout; air can only reach them on one side. The passages are so narrow that sunlight never enters many dwellings.

There are underground rooms here. These are infested by rats, yet people sleep in them. They sleep on the stairs, they sleep under the stairs. The door in a vast number of houses opens into the living-room, one step down. Heavy rain washes the street dirt into this room continually.

Some such houses, condemned many years ago, have stood till, tired of waiting to be demolished, they have fallen down. The front wall has fallen out, the landlord has re-built it; things have gone on as before.

Alien immigration has added to the trouble. A whole street has sometimes been bought up, the inhabitants turned out, and the houses filled with the poorest foreigners. Sweating, and Sunday trading, leaving no day of rest, have come with them. The speech from the Throne promised measures against the "dumping" of goods. What of dumping *people*? Mr. Edmonds knew one room where eleven people lived day and night. He knew recently of one inhabited by eight. If one were ill, work went on in the room, people had to eat and sleep. If one died the coffin might balance the coalbox on either side of the fire for a day or two. *But the English population of Bethnal Green sent proportionately more men to save the world from the Hun than any other borough of London.* Landlords deserve no capital sum in compensation for rents (—— wicked rents, said Mr. Douglas Eyre) for such dwellings. Let them have the value of the site and such value as there may be in the building materials.

Now we cannot plead ignorance. What is to be done? "People cannot be turned into the street while measures are taken." We put up tents, and then huts, for our soldiers, who had to be kept in health, and we commandeered empty houses in every town. We can, if we choose to take as much trouble to fight the germ as the German, if we think it worth while to carry on the Christian war against disease and vice, do the same for the people, who ought not to spend one more hour in such surroundings. Shall we? Or shall Bethnal Green still cry, "O Lord, how long?"

